# Corporate practices and health: a framework and mechanisms

**DOI:** 10.1186/s12992-018-0336-y

**Published:** 2018-02-15

**Authors:** Joana Madureira Lima, Sandro Galea

**Affiliations:** 1 Department of Sociology, University of Oxford, Manor Rd Building, Manor Road, Oxford, OX1 3UQ UK; 2 School of Public Health, Boston University, 715 Albany St, Boston, MA 02118 USA

**Keywords:** Non-communicable diseases, Transnational Corporations, Macrosocial Determinants of Health, Corporate Influence, Corporate Tactics

## Abstract

**Background:**

The Global Burden of Disease estimates that approximately a third of deaths worldwide are attributable to behavioural risk factors that, at their core, have the consumption of unhealthful products and exposures produced by profit driven commercial entities. We use Steven Lukes’ three-dimensional view of power to guide the study of the practices deployed by commercial interests to foster the consumption of these commodities. Additionally, we propose a framework to systematically study corporations and other commercial interests as a distal, structural, societal factor that causes disease and injury. Our framework offers a systematic approach to mapping corporate activity, allowing us to anticipate and prevent actions that may have a deleterious effect on population health.

**Conclusion:**

Our framework may be used by, and can have utility for, public health practitioners, researchers, students, activists and other members of civil society, policy makers and public servants in charge of policy implementation. It can also be useful to corporations who are interested in identifying key actions they can take towards improving population health.

## Background

*“We learn that your country is a 60 or 7000 lee away from China and yet foreign vessels come here to make great profit out of the wealth of our country. But by what right, in return, do they sell us this poisonous drug that does so much harm to the Chinese people? They may not necessarily intend to hurt us, but by putting profit above all things they are disregarding the harm they do to others”.*

Commissioner Kin to Queen Victoria soon before the start of the first Opium War [1820–1840].^1^.

The Global Burden of Disease estimates that approximately a third of deaths worldwide are attributable to behavioural risk factors, including alcohol, drug and tobacco use, and poor dietary profile. Underlying these risk factors is a common aetiology: overconsumption of unhealthful products and exposures all of which are, essentially, produced by commercial entities. It therefore seems apposite to consider the role these commercial entities play in shaping their products, driving consumption, and in turn, influencing population health.

Naturally, not all corporate products are unhealthful. Lifesaving medical technologies and medicines are produced and distributed by corporations, as are some high nutrient-low calories content foods. Furthermore, in recent years, demand for more corporate responsibility has increased and numerous companies have stated their commitment to a “Triple Bottom Line” approach of People, Planet and Profit. The operative question therefore is: how can we better understand corporate practices to the end of promoting salutary practices and minimize negative consequences of corporate influences on health.

Research in this area has documented corporate practices of influence in particular industries such as tobacco [1–6], alcohol [7–12], food and drink [13–17], chemicals [18–21], automobile, weapons, extractive industries and pharmaceuticals [22–26]. Another field of inquiry has shown commonalities across corporate behaviour in different industries [27–31].

What is missing from this analysis is an overarching theoretical framework to systematically study commercial interests as distal, structural, societal factors that cause disease and injury. This framework will help guide the study of these practices in the wider social, political and economic context [27].

A comprehensive framework is needed to ensure that no aspect of corporate action is left unexamined by academics and practitioners alike. For example, in their otherwise thorough framework for conducting corporate health impact assessment Baum and colleagues do not mention how corporations may interfere with the research process and redefine what constitutes scientific evidence for policy making. Neither do they include a measure of how corporations engage in changing the social discourse around the role of the state or the social value of regulation [32, 33].

**Fig. 1:**
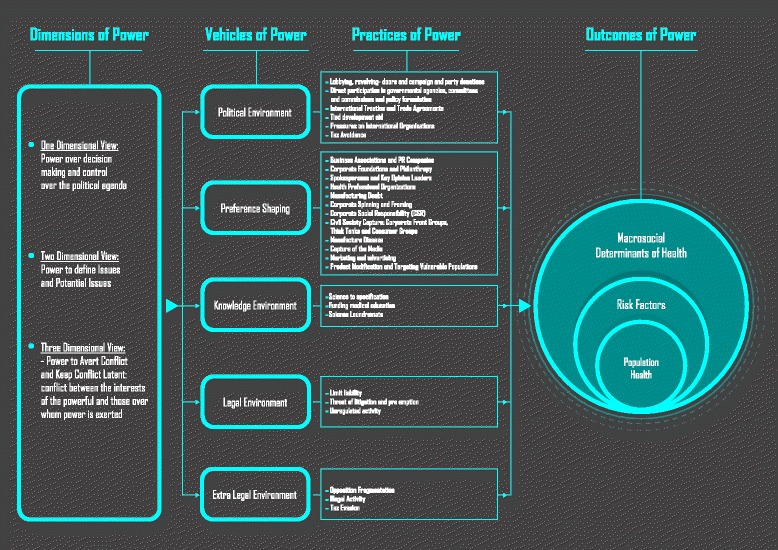
Diagram of Dimensions, Vehicles, Practices and Outcomes of Power

We put forward such a contribution using the work of Steven Lukes as a starting point. Lukes proposes a three-dimensional view of power that builds on the one and two-dimensional views of his predecessors. The study of these dimensions can be guided by a focus on four features of power. The One-Dimensional view focuses on Power over decision making and control over the political agenda [not necessarily through decisions]. The Two-Dimensional View focuses on Power to define what constitutes an issue and a potential issue. The Three Dimensional View expands its focus to encompass the Power to avert observable conflict [overt or covert] and Power to keep conflict between the interests of the powerful and those over whom power is exerted latent [34]. [See Fig. 1].

The power lens structures our thinking and inquiry around the practices through which commercial power is exerted and ultimately, how population health is influenced. To this end, we suggest five vehicles through which each of the Dimensions of power express themselves, as well as the specific practices that enable that expression. The One Dimensional View of Power focuses on decision-making, and how influencing decision-making exerts control over the political agenda. The Two Dimensional View refers to the ability to keep issues out of the political and social spheres by shaping public perceptions, cognitions, and preferences in such a way that no other options are deemed possible or desirable. The Three Dimensional View extends its focus beyond that of the One and Two Dimensional Views to encompass the aversion of conflict over issues that may harm the interests of those in power. The aversion of conflict results in latent conflict between the interests of the powerful and those of society at large, often to the detriment of the latter.

Power, in its three Dimensions, may be exerted through five vehicles that we named as follows: the Political Environment, Preference Shaping, the Knowledge Environment, the Legal Environment and the Extra Legal Environment. These vehicles will find their expression through technical means of implementation which we call Practices of Power. Practices of Power are the tools that corporations use to operationalize their agenda. They include, for example, lobbying activities and campaign donations as well as illegal activities. The distal outcome of the exertion of power is an unbalance in the macrosocial determinants of health, which in turn, have an effect in the incidence of risk factor for disease. The proximal outcome is a deterioration of population health.

In the rest of this paper we discuss what constitutes each of the vehicles of power and how they find their expression through examples of the use of different enablers.

### Political tactics: State and institutional capture

Control over decision-making, agenda-setting, and non-decision allows commercial actors to keep statutory regulation at bay and to shape the narrative and public perceptions around the role that governments should play. Regulation can be averted through deregulation and non-enforcement of existing legislation [4], the promotion of self-regulation with the adoption of voluntary codes and non-regulatory initiatives [35], delaying implementation of existing regulations, and the perpetuation of loopholes that make tax avoidance legal and tax evasion go unpunished. Changing public perceptions around the role of government as a regulator can be achieved through support for candidates and parties who endorse reduced government participation in public life; through watering down of regulatory powers via direct participation in regulatory bodies and through support of models of international trade that perpetuate historical power differentials in ways that leave little or no room for reducing health inequalities across and within countries. We discuss below the vehicles through which political tactics consolidate the influence of corporations and their potential impact on health.

#### Lobbying

Lobbying confers undue influence on corporate actors for two reasons. First, corporations invest enormous amounts of money in lobbying. The food industry, for instance, spent $1.5 billion lobbying against the European Union’s [EU] adoption of the traffic-light approach, opposing most aggressively the use of a red light suggesting that “any food was too high in anything” [36]. This gives corporations access to policy makers unavailable to regular voters and public interest organisations. Corporate resources invested in lobbying far outweigh those invested by other civic sectors who might advance a more balanced public discussion about societal investment that may ultimately shape health. For example, in 2015 the EU Transparency Registry reported 177 full-time lobbyists working on pharmaceuticals issues on behalf of the pharmaceutical industry versus the 48 advocating on behalf of civil society [37]. Second, the information that corporate lobbyists share with government officials carries weight as expert information, even though it may be biased, incomplete, or erroneous [17, 38].

#### Revolving doors

The flow between public and private sector employment may advance corporate interests in three ways. First, it may result in policy decisions about products and practices that favor industry interests [17, 38, 39]. Second, it guarantees industry a voice in the policymaking process, even though other stakeholders have no assurance that their concerns will be addressed by regulatory agencies [40]. Thirdly, it gives industry a competitive advantage when former regulators bring confidential information into their corporate roles. For example the European Medicines Agency has come under scrutiny when it surfaced that a number of high ranking officials, including the Director, were either previously employed in or moved on to the pharmaceutical industry after employment at the Agency [41–43]. Concerns were raised over the objectivity of these individuals in regulating, for instance, publication and access to clinical trials data [44].

#### Campaign and party donations

Corporate donations enable favourable decision-making and political agenda-setting because reciprocity may be expected once the party or candidates are in office. In the US, for instance, corporations have acquired 1st Amendment rights and can make unlimited contributions to election campaigns directly from the corporate treasury [45]. These donations have observable effects on health policy. In 2003 the US House of Representatives voted on the Pharmaceutical Access Act that would have allowed US retailers to buy the same drug from countries offering lower prices. The bill never became law. Evidence shows that the coalition to block drug re-importation received more cash than the coalition to allow re-importation and that the pharmaceutical industry used campaign contributions to reward or punish each representative’s vote. Whilst other factors may explain voting patterns, pharmaceutical campaign donations were found to be the most important explanatory variable [46, 47].

#### Direct participation in governmental agencies, committees and commissions and partnerships with government for policy delivery

Companies whose interests may conflict with the public’s may protect and expand their activities by becoming partners in the formulation of public policy [48]. The US Department of Agriculture not only receives financial contributions from agribusiness, but also, historically, has had staff and agriculture committee members from the farming community - particularly meat producers [49–51]. This is an important factor explaining why the antibiotic use for meat growth – a driver of antimicrobial resistance- is left to be voluntarily regulated by the meat and pharmaceutical industries as opposed to statutorily regulated by government agencies [49, 52, 53]. Similarly, the UK’s Department of Health anti-obesity marketing campaign Change4Life was drafted by a coalition of food and drink industries whilst the campaign marketing, paid for by the obesity budget, was awarded to companies which had processed and fast food clients in their portfolio. Moreover, industry was allowed to contribute to the campaign in kind which led to the introduction of obesogenic foods into schools [11].

#### Commercial pressures on international trade negotiations

Commercial pressures may shape population health via trade agreements in three ways: increased availability and decreased prices of unhealthful commodities, increased patent protection of essential medicines via bilateral trade agreements, and decreased levels of protection for occupational health and the environment.

The first mechanism is a consequence of the removal of barriers to imports and FDI. Increased penetration of supermarkets in food retail, for instance, facilitates a shift towards the consumption of processed foods [54, 55]. Furthermore, FDI by multinational food companies has driven an increase in availability of processed food in developing countries [56].

Through the second mechanism, trade agreements between two countries eliminate WTO provisions that protect public health under multilateral agreements. For example, under the Agreement on Trade-Related Aspects of Intellectual Property (TRIPS), low income countries may invoke flexibilities allowing them to produce generic versions of patented medicines in a public health emergency, which may cause large pharmaceutical companies to lose revenues. Since 2001, every trade agreement signed or under negotiation by the US has increased the terms and scope of intellectual property right protection of pharmaceuticals, including patent terms beyond the 20 years provided for under the WTO eroding these hard-fought flexibilities [57].

In the third mechanism, pressures on the international trade architecture have ensured that adherence to labour standards is not a requirement either in trade agreements administered by or a condition for membership in the WTO. In fact, article XX, often referred to as the escape clause, outlines when members do *not* have to fulfil trade commitments and that covers public health and environmental standards [58].

#### Tied aid

Large corporations shape population health in the developing world by lobbying governments to formulate development aid so to increase their participation in it. Tying food and medicines aid to purchases from corporations in donor countries acts as de facto export promotion and overcharges recipient countries for commodities they could procure elsewhere at lower prices [59, 60]. Consequently, excess crops are dumped in developing country markets reducing local food production and bringing down food prices for local farmers [61], new markets for food exports open [62] and competition from developing economies eliminated. In 1986 strong lobbying efforts by the American Soybean Association resulted in the passage of the “Bumpers Amendment” by the US Congress so that no financing, technical assistance or capacity building should be delivered to crops that might compete with US exports and assistance of any kind should be directed at palm or coconut oil, so not to compete with US soy oil. Soy oil is one of the substrates for the production of trans fatty acids whose consumption is associated with increased risk of heart disease [63].

#### Pressures on international Organisations

Commercial players may protect their interests via their governmental representatives or directly via participation in delegations to international bodies with mandates to regulate their activities. During the 1970s, the International Labour Organisation, the Organisation for Cooperation and Development and the United Nations Centre on Transnational Corporations each attempted to create mandatory codes of conduct for corporations. The former two complied with the call of business to create suggestive codes without penalties for non-compliance and the latter never achieved consensus to adopt its recommended accountability structures [64]. More recently, the Sugar Association, representing the U.S. sugar industry, was critical of a WHO report on guidelines for healthy eating, which suggested that sugar should account for no more than 10% of a healthy diet. The association demanded that Congress end its funding of the WHO unless it withdrew the guidelines. Moreover, the association and six other big food industry groups also asked the U.S. Secretary of Health and Human Services to use his influence to get the WHO report withdrawn [13, 65].

### Preference shaping: perception, cognition and need creation

Power over preference shaping is exerted not only by fostering preferences for particular products and lifestyles but also for the role of personal responsibility, and that of the government, in mediating the public’s interactions with these products.

Commercial entities exercise power over preferences by ensuring that tensions between the interests of population health and theirs are never constructed as political or social issues. For example, if a harmful product is portrayed as safe, healthful, and even necessary, as long as the consumer perceives it as such, there will be no need to confront the manufacturers about its toxicity. Accordingly, we cannot take an absence of health grievances to mean that there is a genuine social consensus around unhealthful commodities. Rather, we should focus on the question of whether that consensus was fabricated by shaping preferences in such a way that grievances never arise [34].

The conflict aversion feature of power ensures that tensions between corporate and public health interests never result in open confrontation. Open confrontation may harm the corporate image and the bottom line and so a number of tactics, such as the deployment of public relations companies or the enlistment of key opinion leaders described below, are used to manage conflict. When it arises, these enlisted actors keep conflict latent or completely avert it when it can be anticipated.

#### Public relations companies

International public relations (PR) companies pool together expertise and financial resources from industries with similar needs in terms of regulation and social acceptability for their products. They mount multi-pronged campaigns that target the media, legislators and consumers to dismiss public health concerns. PR companies working for the supplements industry in the US to fight against stricter regulations by the FDA built a comprehensive campaign. It included questioning the Agency’s mandate to “tell citizens how to choose their diet”, the portrayal of regulatory action as prohibition of health enhancing supplements and convincing consumers of the benefits of these substances despite the lack of scientific evidence [51].

#### Key opinion leaders [KOL] and funding health professions organisations

Through enlisting key opinion leaders, commercial interests may shape the background of accepted issues and opinions in a given field and prepare the target audience to welcome new drugs, technologies and diagnosis. Researchers on female sexual dysfunction held industry sponsored workshops and wrote position papers that solidified female sexual dysfunction as an illness, thereby positioning themselves as the very experts to whom the FDA would turn on for advice on drug submissions and to whom the media would turn to for interviews and information [66].

#### Manufacturing doubt

Corporations may avoid both a negative image and statutory regulation of their products by casting doubt on the scientific evidence documenting negative effects associated with them and by discrediting the scientists who produce such evidence. The rationale is that if the existing evidence around the harmful effects of a given product is ambiguous and there is no consensus around it, then there is no need for regulatory action [67].The cases of tobacco, acid rain, ozone, global warming, and DDT all have this tactic in common: a group of scientists with extensive political connections, ran effective campaigns to mislead the public and deny well-established scientific knowledge over four decades [68].

#### Corporate foundations and corporate social responsibility [CSR]

There are two ways in which CSR increases exposure to harmful corporate products that would otherwise be more tightly regulated. Firstly, the voluntary nature of CSR prevents its meaningful formalization into statutory regulation [15, 64] removing incentives to protect public health. This is an explicit corporate strategy: “Companies should not allow social responsibility to divert their attention from their main goal, which is to maximize shareholder value…Behind the pressure to adopt social responsibility there is a profit motive. Putting people before profits is wrong tactic” [69].

Secondly, CSR expands corporate marketing reach and product acceptability by associating it with an image of social commitment. The tobacco industry engaged in youth smoking prevention programmes as part of its CSR in North and Latin America. Even though there is no evidence that these programs reduce smoking among youths, they have met the industry’s goal of portraying the companies as concerned corporate citizens. What is more, they undermined effective tobacco control interventions such as those outlined in the WHO Framework Convention on Tobacco Control [70]:among youths, each additional viewing of a tobacco company parent-targeted advertisement is, on average, associated with lower perceived harm of smoking [71].

#### Global Health philanthropy

Philanthropic institutions may act as channels for unhealthful corporate products in two ways. First, foundations may extract revenue from products that are harmful to health. Stuckler and colleagues found that Bill & Melinda Gates Foundation’s [BMGF] corporate stock endowment is heavily invested in food and pharmaceutical companies, directly and indirectly. For example, it holds significant shares in McDonald’s and Coca-Cola [72]. The stock on major determinants of NCDs –highly processed foods and sugary drinks- deserves attention given the low priority the Foundation gives to NCDs, despite their growing importance in the global burden of disease.

Second, through their expanding role in financing supranational organizations such as WHO, WB, GAVI or the Global Fund, these foundations have a say in setting international health policy priorities. The funders’ priorities are often driven by personal interests and not necessarily aligned with the health policy priorities of the recipient country [73]. The BMGF’s portfolio of pharmaceutical companies calls for attention given Mr. Gates’ personal belief in the role of patents as motors for innovation in medicines and medical technology. Activists in the access to medicines movement have publicly criticised this stance [74] given his involvement in initiatives such as GAVI or the Global Fund that rely heavily on relationships with the pharmaceutical industry to secure access to drugs.

#### Civil society capture

The corporate co-option of grassroots formations - e.g. consumer and patient groups, or research organisations – e.g. think tanks - prevents public health issues from arising and averts conflict over them in three ways. It confers legitimacy to industry claims, it deflects criticism from public health advocates and serves as a media response tool.

For example, the National Smokers’ Alliance was developed by Burson-Mersteller, a PR company, on behalf of Philip Morris, and the Californians for Realistic Vehicle Standards is a front for the auto industry opposing air pollution regulation [18]. Under the guise of a patient constituency, for instance, pharmaceutical industry founded and funded patient advocacy organisations may pressure third party payers to pay for certain drugs regardless of efficacy or efficiency or regulatory agencies for drugs to enter the marketplace more quickly despite the potential harms of an expedited review [75, 76]. Health and patient organisations lobbying the European Institutions who receive funding from the pharmaceutical industry include the European Kidney Health Alliance, the European Respiratory Society and the European Union Geriatric Medicine Society [37].

Corporate funded think-tanks are the top down counterpart to the “grassroots” movement. These institutions operate under the veil of academic independence to lead public opinion and set the terms of public discussion. In the first year of the Bush administration alone, out of the 44 pieces of regulation contested by Mercatos, a think-tank funded by the libertarian Koch brothers, 66% were selected for elimination of modification [18].

#### Issue framing and attention deflection

Framing public health issues in terms of personal responsibility for making informed choices takes the onus away from the harmful composition of certain products, from their availability, marketing and advertising and ultimately from their regulation. The automobile industry has long avoided stricter regulations by shifting responsibility to the driver and claiming that the driver, not the vehicle nor the built environment in which it operates, is the problem. This rhetoric was documented in vehicle safety regulation, alcohol blood levels, speed limits, mandatory seat belts and airbag regulations, etc. [77].

Promoting actions outside the corporation’s area of expertise deflects criticism from its main line of business. Tobacco companies have promoted campaigns to prevent violence against women and funded shelters for victims of domestic abuse [13, 78]. Similarly, the tobacco and alcohol industries have engaged in activism and advocacy against the smuggling of alcohol and tobacco products as a means to avoid regulation of their activities [6]. For example, cigarette smuggling is often cited as an argument against a tobacco tax increase [79] even though the tobacco industry engages in the smuggling of its own products to avoid duty tax [4].

#### Manufacturing disease

By creating new diagnostics and thresholds for medical intervention, commercial interests expand the consumer base and reduce the scope for less profitable population wide approaches for tackling diseases rooted in social and environmental problems. This may be done in two ways. One is to expand diagnostic criteria to include what were previously considered normal laboratory results, behaviours or feelings [80, 81]. The financial ties between DSM-V panel members and the pharmaceutical industry have shed light on this mechanism [82, 83]. More encompassing diagnostic criteria are then complemented by an effort to increase acceptability of a given condition. This is evidenced by the penetration of the pharmaceutical industry into the education sector by approaching teachers to diagnose Attention Deficit and Hyperactivity Disorder [84].

When diagnostic guidelines are not yet available, new conditions may be created by pathologizing complex behaviours and promoted directly to the public, as was the case of female sexual dysfunction [85].

#### Capturing the media

The media has the power to mould perceptions of product acceptability and desirability and to shape the discourse around the role of government, personal responsibility and accountability of private interests when their products harm health. Corporations harness this power through media ownership, control over advertising as a lever over editorial decisions, and by using the media to influence jurors on litigation cases against harmful products. Together, these tactics hedge against the coverage of harmful stories and secure favourable treatment of corporate activities and image. When the tobacco industry levered its advertising clout to avoid coverage of anti-smoking stories it enlisted newspapers’ backing against restrictions on tobacco advertising, a crucial source of revenue [18].

#### Marketing and advertising

Combined, marketing and advertising expand the number of consumers for a given product and shape the psychological and social predisposition to accept and endorse hyperconsumption. Advertising strategies have drawn on advances in neuropsychology, anthropology, and neuroscience to capitalise on the susceptibilities of a developing brain to marketing messages [69]. Point in case, neuromarketing uses physiological and clinical information about brain functions to bypass evaluation processes underpinning decisions about what to buy that came about through millennia of evolution [31]. The yield of these technical advances is then complemented by a consonant consumption environment. For example, well-funded marketing strategies are applied to corporate social responsibility, as it becomes the only source of health education in some settings, namely in more economically deprived environments [30]. These compete with underfunded, yet evidence based public health campaigns.

#### Product modification and targeting vulnerable populations

Markets in traditional consumer demographics in the developed world have reached saturation, partly due to tighter regulation. Consequently, multiple industries have turned to the developing world where disposable incomes are increasing and regulations are still weak to expand its consumer base [16, 86, 87].

Others expanded into previously untapped markets, e.g. young people [9], women [88] or problem drinkers. Low income groups, for example, are a particularly profitable demographic to the alcohol industry. This is partly why it resists attempts to raise the price of alcohol, be it through minimum unit pricing or by increasing excise taxes, measures shown to discourage drinking [10]. Low prices allow industry to sell greater quantities in low income groups, who traditionally have shown higher prevalence of alcohol abuse.

### Shaping the knowledge environment

The breadth and depth of knowledge about corporate products and how they affect health bears directly on consumption, perceived need for consumption, and corporate image. Accordingly, corporations carry out scientific work with the express purpose of reaching a conclusion that supports industry regulatory or litigation objectives as well as providing scientific underpinnings for a commercial venture such as a new drug [18].

Seen through a power lens, commercial interests set the research agenda, define what is studied, and thus what constitutes an issue and what does not. The power to constitute non-issues, in turn, generates power to avert conflict over products and processes that harm health. In other words, if harmful effects of corporate products are not systematically documented and attributed to that product, ascription of responsibility will not trigger conflict. Should conflict arise nonetheless, power over research interpretation and dissemination keeps it latent. As long as conflict is averted or kept latent there is no threat to the bottom line.

#### Control over the research process

Three tactics enable corporate control over the research process. First, the funder sets the research agenda by funding research at universities, in-house corporate laboratories, non-profit research institutes or for-profit science-for-hire firms. Second, funding allows control over study design, analysis methodology and ownership of the data. Corporations funded £250 million of university research in 2001 in the UK. In the US, private commercial funding grew from $264 million in 1980 to $2 billion in 2001 [19]. Naturally, corporate funding, per se, is not a problem but there is substantial empirical evidence that the independence of the research is compromised by it. An analysis of published randomised control trials in 12 specialties showed that financial competing interests were significantly associated with authors’ conclusions [89, 90]. The selective reporting of studies funded by the pharmaceutical industry to regulatory agencies was found to be the major cause of bias in estimates of a drug’s efficacy and safety when these studies were submitted for approval of new indications for existing drugs [91]. Furthermore, conclusions of meta-analyses [92] and cost effectiveness analysis [93] have also been shown to be biased towards the funding industry’s interests.

In the third mechanism, the funders may suppress or misreport unfavourable results through exclusive ownership of the data [24, 25, 94]. Access to unpublished trial reports submitted to drug regulatory agencies sheds light on possible side effects of the drugs because they provide more reliable data than published papers. Editors, peer reviewers, and editorial writers who are trusted to evaluate the accuracy of the analyses are thus often unable to do so [95]. Consequently, side effects that are known only to the sponsor result in avoidable deaths, as evidenced by the Vioxx [Rofecoxib] scandal. Rosiglitazone’s withdrawal from the market illustrates yet another layer of control over the data: GSK had direct access to the Data and Safety Monitoring Board - in charge of monitoring data being collected in clinical trials. The fact that commercial sponsors of the trial have direct access to these bodies, thwarts due course and prevents them to stop trials that endanger participants [96].

#### Funding medical education

Funding of symposia, hospital lectures, and medical specialty meetings ensures that the educational content is shaped to favour certain products and procedures over others, without mandatory scrutiny of claims of superiority. The recruitment of KOL lends scientific and pedagogic credibility to the financial efforts. These unfiltered exposures fulfil the double objective of influencing prescription patterns and shaping the medical community’s attitude towards critically appraising what counts as evidence [97].

#### Scientific advisory boards [SABs] and science institutes

This last tactic pertains to image control once health damaging effects of corporate products are made public. SABs are organized groups of “industry friendly” “third party” scientists who support industry’s scientific positions in regulatory processes, the courtroom and public opinion [18]. Tobacco companies established the Centre for Tobacco Research Advisory Board [13] and beryllium companies established the Beryllium Industry Scientific Advisory Committee [18]. There is no requirement for these companies to disclose their funding when lobbying regulatory agencies or when expressing their views in other public fora. Science Institutes fulfil a similar role [19]. The Asbestos Information Association is a front group created by this industry to dismiss public and governmental concerns about their product. It has a successful track record of weakening regulations coming from the Occupational and Safety Health Agency and the Environment Protection Agency in the United States [18].

### Legal systems and the law

A strategic use of the law can lower barriers to undue influence over the political process - e.g. giving corporations personhood - thereby facilitating political agenda-setting and decision-making. This prevents contentious public health protection issues from ever being brought to the legal system, thus averting open conflict between corporations and those advocating for public health.

Influence over judicial systems may stifle legislative attempts to protect against harmful products and practices or water down more comprehensive occupational or environmental health legislation. It can also ensure that sanctions for harming population health are not enforced. Both instances are profitable because they allow, among other things, for a legal mechanism to externalise costs.

Finally, when the interests of commercial players are in open conflict with those of population health and are brought to trial, the legal system may be used in such in a way that conflict is never resolved. This is beneficial from a corporate perspective because the product or practice in question remains available, profitable, accountability never ascertained and corporate image intact.

#### Liability

Corporations protect themselves against accountability for wrongdoing by changing the law and reinterpreting its spirit and intent. By averting liability, corporations avoid negative associations with their brand, reparations and regulation that may curtail further exposure to their unhealthful practices and products. In the US, 4th Amendment rights confer corporations the right to privacy and thus enable avoidance of health and environmental inspections. In 1986 corporations claimed the right to refuse surprise inspections by the US Environment Protection Agency and the Occupational Health and Safety Administration severely limiting these agencies scope for health protection [64].

Corporations may also have an unlimited lifespan and limited liability of shareholders for corporate practices [69].When a corporation is treated as a person under the law, it is liable for its actions. This means that its directors and managers may not be held responsible for any wrongdoing in which the company has engaged [98]. This eliminates the dissuasive effect that may deter harmful practices.

#### Threat of litigation

Corporations use the possibility of costly and time-consuming litigation to deter action that may bring the public’s attention to unhealthful products and practices. Litigation may be used as retaliation, as in the case of the McDonalds’s lawsuit against British activists [99], or as a deterrent for legal action. The lawsuits that Philip Morris brought against Uruguay over graphic cigarette health warnings [100] and against Australia over plain packaging, are warning signs for countries considering similar legislative attempts [101]. This is may be an especially strong message against regulatory initiatives for poorer countries with fewer resources.

#### Externalize costs using unregulated areas of activity

Corporations may keep prices of harmful products artificially low and more available when the final price does not reflect the full cost of production. This is the case with environmental and occupational health costs when they befall on taxpayers. In a practice known as “tolling”, for instance, a manufacturer sends its chemical product to another company to have it processed, maintaining ownership of the product, but not responsibility for occupational health [18]. Similarly, the meat industry has long kept meat prices low by avoiding the environmental, social and occupational health costs of large concentrated feeding operations for animals raised for food [102–104]. What is more, externalizing occupational health costs is effectively sanctioned by the international trade architecture as pointed out in section 1.5.

### Extra legal tactics

When, despite efforts to influence decision-making and agenda-setting, non-issues become public health issues, open conflict over those issues arises and corporations may break the law in order to secure their interests. Of all corporate criminal offenses prosecuted by the US Department of Justice between 1974 and 1976, for instance, 60% occurred in just three industries, all of which directly relevant to population health – petrochemicals, pharmaceutical and automobile manufacturing [105].

#### Fragmentation of opposition groups

By discrediting public health advocates’ opposition or portraying the public health community as fragmented, commercial interests ensure that there is no countervailing discourse. Phillip Morris’ Project Sunrise explicitly aimed at fragmenting the anti-tobacco movement by dividing advocates into “extremists” and “moderates” and building partnerships with the latter, which brought them some legitimacy [106]. Direct tactics include corporate infiltration of opposition groups [105, 107] as in the case of corporate infiltration of anti- bovine growth hormone advocacy groups [50].

#### Corporate illegal activity

This category includes bribing [108], smuggling and illicit trade [4, 109], and price fixing [110] among others.

When these offenses are prosecuted, out of court settlements elude corporate accountability. Corporations avert criminal prosecution and the ensuing fines are still financially attractive to companies for whom the penalty is only a small fraction of annual profits. In 2009 Pfizer paid what at the time was the largest criminal fine ever imposed in the US ($2.3 billion) in an out-of-court settlement for the off label promotion of several drugs only to be surpassed in 2012 by GSK with a $3 billion settlement after pleading guilty to unlawful promotion and failure to disclose drug safety data [111].

## Conclusion

Corporate activities have immediate and observable effects on perceptions and behaviour patterns that can lead to increased consumption of unhealthful corporate products and subsequently to changes to individual and population health. We caution that this should not be interpreted as suggesting that corporate activities are necessarily harmful to health, nor that there is anything untoward about most corporate action, which with few exceptions, operates well within legal and culturally acceptable bounds. Our intention in critically and systematically studying the mechanisms of corporate action is to highlight how corporations may influence population health to the end of mitigating corporate action on poor health.

Our framework offers a systematic way of mapping corporate action as a way of guiding research and practice. It is meant to be used by public health practitioners, researchers, students, activists and other members of civil society, policy makers and public servants in charge of policy implementation. It can also be useful to corporate managers who wish to establish or improve triple bottom line principles.

## Footnotes

1 Wood [112]
